# Prevalence of pain and associated factors in Brazilian civil servants: an introductory analysis using baseline data from the ELSA-Brasil cohort

**DOI:** 10.1097/PR9.0000000000000797

**Published:** 2019-12-06

**Authors:** Luciana A.C. Machado, Rosa W. Telles, Isabela M. Benseñor, Sandhi M. Barreto

**Affiliations:** a University Hospital, Universidade Federal de Minas Gerais (UFMG), Belo Horizonte, MG, Brazil,; b Faculty of Medicine, UFMG, Belo Horizonte, MG, Brazil,; c University Hospital, Division of Internal Medicine, Universidade de São Paulo (USP), São Paulo, SP, Brazil

**Keywords:** Pain, Prevalence studies, Epidemiology, Developing countries

## Abstract

**Introduction:**

In Brazil, the prevalence and costs of pain will increase substantially with population ageing. Understanding of pain epidemiology is needed for the development of health care policies that can minimize this projected burden.

**Objective:**

To investigate the prevalence of pain and associated factors at baseline of the Brazilian Longitudinal Study of Adult Health (ELSA-Brasil).

**Methods:**

Data were collected in public institutions of higher education/research (2008–2010). Pain in the past 30 days and pain attributed to psychological distress (“with psychological attributions”—PPA) were evaluated by the Clinical Interview Schedule-Revised (CIS-R). The independent *t*-test and χ^2^ test investigated associations between sociodemographic/clinical factors and each pain episode. Multivariable analyses including age, sex, leisure-time physical activity, depression, and arthritis/rheumatism, and factors showing univariate associations at the *P* < 0.10 level, were performed.

**Results:**

Fifteen thousand ninety-five civil servants were included (52.1 ± 9.1 years, 54.4% female). The prevalence of any pain was 62.4% (95% confidence interval 61.6%–63.2%), and of PPA was 22.8% (95% confidence interval 22.2%–23.5%). Factors associated with any pain and PPA in multivariable analyses included age (odds ratio [OR] 0.97), female sex (OR 1.86–2.01), moderate and vigorous leisure-time physical activity (OR 0.60–0.84), excessive drinking (OR 0.68–0.83), depressive symptoms (OR 1.28–1.96), anxiety symptoms (OR 1.63–2.45), sleep disturbance (OR 1.62–1.79), and arthritis/rheumatism (OR 1.32–2.18). Nonroutine nonmanual occupation (manual occupation as reference), body mass index, and smoking were independently associated with either any pain or PPA.

**Conclusion:**

This study provided preliminary information on the epidemiology of pain at baseline of the largest Latin American cohort on chronic noncommunicable diseases.

## Abstract 2

Supplemental Digital Content is Available in the Text.

## 1. Introduction

There has been an increasing awareness of the importance of chronic pain for public health on a global stage.^13,48^ Chronic pain is generally defined according to its duration, as pain that persists for 3 or 6 months.^38^ According to Global Burden of Disease (GBD) studies, painful musculoskeletal conditions are among the commonest chronic noncommunicable diseases (NCDs) worldwide, and some of these conditions (eg, low back pain) have been consistently ranked over the last 20 years as the top contributors to years lived with disability in both men and women.^26^ Although previous GBD studies have not considered chronic pain in its own right until this date, data provided for musculoskeletal conditions reflect, at some extent, the global burden of chronic pain.^11,12^

Findings from recent meta-analytic summaries on the prevalence of chronic pain in regions with distinct development levels indicate that over one third of individuals will suffer from this condition at any given time.^24,32^ In low- and middle-income countries, the prevalence of chronic pain has been estimated at 33% in the general adult population, 35% among workers, and 56% among elders.^32^ The faster pace of demographic transition in these less developed nations is of great concern because they may not be able to implement efficient public health policies and/or increase health care services availability for NCDs (including chronic pain) at a rate as fast as population ageing.

Over the last 3 decades, Brazil has faced a rapid increase in life expectancy and longevity of its population due to a sharp decline in transmissible-diseases mortality risk, maternal-infant morbimortality, and avoidable causes of death.^18^ Nevertheless, there is still a paucity of relevant information regarding the burden of chronic pain in Brazil, particularly due to the lack of high-quality epidemiological research; eg, most of this research has shown to carry a moderate-to-high risk of bias.^42,43^

Information about the presence of any pain and the characteristics of a pain episode attributed to psychological distress was collected within the framework of the evaluation of common mental disorders in the Brazilian Longitudinal Study of Adult Health (ELSA-Brasil). A preliminary study was conducted to estimate the prevalence and associated factors of pain at baseline of the ELSA-Brasil cohort.

## 2. Methods

### 2.1. Design and participants

A cross-sectional observational study was performed using data collected at baseline of ELSA-Brasil, which is a prospective multicenter study developed by Investigation Centers located in 6 Brazilian states (Bahia, Espírito Santo, Minas Gerais, Rio de Janeiro, Rio Grande do Sul, and São Paulo).^6,50^

The ELSA-Brasil cohort is constituted of active or retired civil servants, aged 35 to 74 years at inception, from 7 public institutions of higher education and research. The required sample size was originally set at 6,400 participants, which was the minimal sample needed for the investigation of the primary aim of the cohort (ie, to study of the incidence of cardiovascular disorders and type 2 diabetes), but the recruitment target was increased to 15,000 participants to account for subgroup differences and loss to follow-up.^5^ Recruitment followed general and local awareness-raising strategies, including the distribution of printed material, development of the study's website (http://www.elsa.org.br) and involvement of the academic community. In addition to those who volunteered to participate, civil servants were also actively recruited from lists of employees provided by the participating institutions. Those with the following characteristics were excluded: intention of leaving the institution, pregnancy or having been pregnant less than 4 months before enrollment, severe cognitive or communication difficulty, and, if retired, living outside the corresponding metropolitan region.^5^

**Figure 1.**
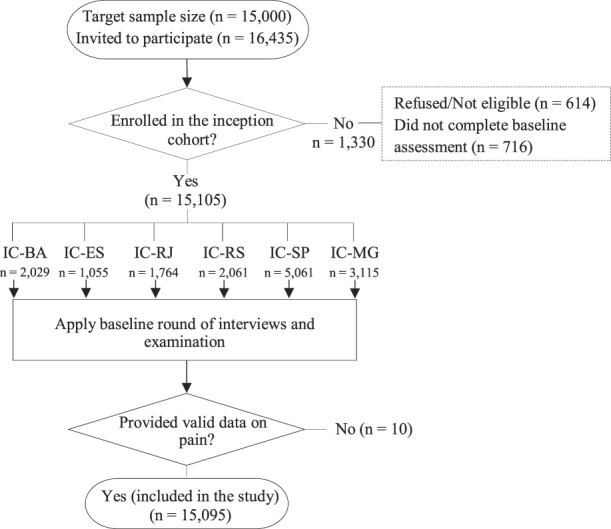
Flowchart for inclusion in this study. BA, Bahia; ES, Espírito Santo; IC, Investigation Center; MG, Minas Gerais; RJ, Rio de Janeiro; RS, Rio Grande do Sul; SP, São Paulo.

At inception, the ELSA-Brasil cohort comprised 15,105 participants. Of these, 15,095 (99.9%) civil servants providing data on pain were considered eligible for inclusion in this study (Fig. 1).

### 2.2. Data collection procedures

Data were collected from 2008 through 2010, in 2 phases: (1) initial interview at the participant's job site, lasting approximately one hour (active workers only) and (2) structured examination at the study clinic (all participants), including face-to-face interviews and exams/tests, lasting approximately 5 hours. Detailed information on data collection and management in ELSA-Brasil has been described in a series of previous publications.^6,9,10,16,23,39,51^ ELSA-Brasil has been approved by institutional ethics committees and the National Committee of Ethics in Research (protocol 976/2006). Participants signed a written informed consent after they had been informed of the nature and details of the study.

### 2.3. Assessment of pain at baseline of ELSA-Brasil

Pain information was retrieved from the section on somatic symptoms (section A) of the Clinical Interview Schedule-Revised (CIS-R), which comprises a questionnaire used for the assessment and diagnosis of nonpsychotic psychiatric conditions.^35^ The complete version of CIS-R has been previously validated for use in the Brazilian population.^45^

The presence of any pain was identified by the question “*Have you had any sort of pain in the past 30 days?*” Participants reporting any pain were also enquired about whether they believed their pain was attributed to depressive feelings, anxiety, or stress through the question “*Was this ache or pain brought on or made worse because you were feeling low, anxious, or stressed?*” Those with a positive answer were considered to have an episode of pain attributed to psychological distress, herein named “pain with psychological attributions” (PPA).

According to the original structure of CIS-R, only participants with PPA were enquired about the frequency of their pain, and only those reporting at least one day of pain in the past 7 days were prompted to answer additional questions about the characteristics of their symptoms: “*In the past week, has the pain been very unpleasant, a little unpleasant or not unpleasant?*,” “*Has the pain bothered you when you were doing something interesting in the past week?*,” and “*How long have you been feeling this pain as you have just described?*” (<2 weeks/≥2 weeks but <6 months/≥6 months but <1 year/≥1 year but <2 years/≥2 years). According to the participant's answers, PPA was characterized as “with negative affect,” bothersome during activity and chronic. Definitions used for each type of pain episode and for the characterization of PPA are listed in Table 1.

**Table 1:**
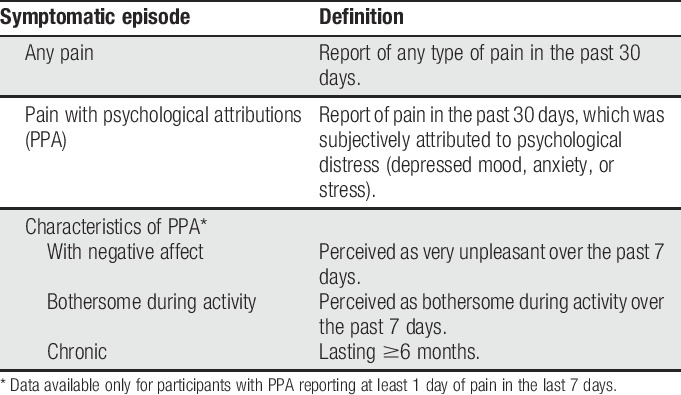
Definitions of pain episodes at baseline of ELSA-Brasil (2008–2010).

### 2.4. Assessment of sociodemographic and clinical factors

Sociodemographic data were collected through standardized assessments. The following variables were considered: age group (35–44, 45–54, 55–64, 65–74 years), sex (male, female), self-declared race/skin color (Black, Brown, White, Asian, Indigenous), work status (active or retired), nature of occupation and occupational social class (current or last if retired), body mass index (BMI) (eutrophic <25 kg/m^2^, overweight 25–29.9 kg/m^2^, obese ≥30 kg/m^2^), leisure-time physical activity (LTPA), smoking status (never smoker, current/former smoker), excessive drinking (>210 g alcohol/week for men and 140 g alcohol/week for women), depressive and anxiety symptoms, sleep disturbance, diabetes, and previously diagnosed cardiovascular disease and arthritis/rheumatism.

Nature of occupation and occupational social class were ascertained in collaboration with economists from the Centre of Regional Development and Planning (CEDEPLAR) of Federal University of Minas Gerais. Nature of occupation was categorized into 3 groups according to definitions proposed by Autor et al.^7^: manual (routine or nonroutine); routine nonmanual; and nonroutine nonmanual. Occupational social class is a summary measure computed by the combination of information on occupation, observed income, and expected income based on the required education level for that occupation (average market value). The latter was calculated according to the Brazilian occupational matrix from 2008 to 2010.^41^ The resulting socioeconomic status measurements were first grouped into 7 strata,^28^ which were collapsed for the present analysis into upper (upper-high + upper-low), middle (middle-high + middle-middle + middle-low), or lower social class (lower-high + lower-low).

Leisure-time physical activity was assessed by the long version of the International Physical Activity Questionnaire (IPAQ)^27^ and categorized as follows: (1) insufficient (no LTPA practice OR some LTPA, but not meeting the other 2 categories); (2) moderate (≥3 days of vigorous-intensity LTPA for at least 20 minutes/day, OR ≥5 days of moderate-intensity LTPA and/or walking, in combination or alone, at least 30 minutes/day, OR ≥5 days of any combination of walking, moderate- or vigorous-intensity LTPA achieving a minimum of 600 metabolic equivalent (MET)-minutes/week); (3) vigorous—vigorous-intensity LTPA on at least 3 days, accumulating a minimum of 1500 MET-minutes/week, OR or ≥7 days of any combination of walking, moderate- or vigorous-intensity LTPA accumulating a minimum of 3000 MET-minutes/week.^31^

Depressive symptoms were assessed by the depression section (section G) of CIS-R, which contains a total of 9 items enquiring about the presence, frequency, and duration of depressive symptoms.^45^ This section begins with 2 introductory questions on overall depressive symptoms in the past month (if participants feel sad or depressed, and if they are still interested in the things they used to do). If one answer is affirmative, additional comprehensive assessment is made regarding symptoms in the past 7 days, with depressive symptoms defined as a score ≥2.^46^ Anxiety symptoms and sleep disturbance were assessed in a similar fashion, by their respective sections in the same questionnaire (sections J and D, respectively).

Previously diagnosed diabetes was identified by a positive answer to at least one of the questions “*Have you been previously told by a physician that you had/have diabetes (sugar in the blood?)*” or “*Have you used medication for diabetes in the past 2 weeks?*” New onset diabetes was identified according to the following thresholds for laboratory values: fasting plasma glucose (≥126 mg/dL), or 2-hour plasma glucose during OGTT (2-hour PG ≥ 200 mg/dL), or HbA1c (≥6.5%).^3,30^

Cardiovascular disease was identified by the report of a previous diagnosis by a physician of coronary heart disease, ie, myocardial infarction or coronary revascularization, heart failure, or stroke. Although a self-reported physician diagnosis of angina pectoris was also included in the assessment of coronary heart disease in ELSA-Brasil, it was not considered because it has been shown to reduce the accuracy of the self-reported cardiovascular disease diagnosis.^58^

Arthritis/rheumatism was identified by a positive answer to the question “*Have you been previously told by a physician that you had/have any of the following diseases: rheumatoid arthritis, systemic lupus erythematosus, rheumatism, 'arthrosis,' arthritis or other joint problem?*”

### 2.5. Statistical analysis

Mean values and SDs (continuous data) and frequencies and percentages (categorical data) were used for descriptive purposes. Prevalence estimates and exact Clopper–Pearson 95% confidence intervals (CIs) were calculated for each type of symptomatic episode (ie, any pain or PPA) in the overall sample. Participants reporting no pain were also coded as nonprevalent cases of PPA.

Independent *t*-tests and χ^2^ tests were used to investigate associations of continuous and categorical sociodemographic/clinical factors with each symptomatic episode in the overall sample and with chronicity (duration of symptoms ≥6 months) in the PPA subsample. Factors showing associations at the *P* < 0.10 level were forced simultaneously into a multivariable logistic regression model (age and BMI were entered as continuous variables). Age, sex, LTPA, depression, and arthritis/rheumatism were entered into all models even if this significance threshold was not reached, given the consistent evidence in the literature supporting their effect on pain. Statistical significance was set at *P* < 0.05 for all tests. All analyses were performed using Stata statistical software (version 14.0; StataCorp, College Station, TX).

## 3. Results

Mean age ± SD of the included participants was 52.1 ± 9.1; 54.4% were female. In the total sample, the prevalence of any pain was 62.4% (95% CI 61.6%–63.2%) and of PPA was 22.8% (95% CI 22.2%–23.5%). Table 2 describes the presence of these pain episodes according to sociodemographic/clinical factors, and the results for the tests of univariable associations.

**Table 2:**
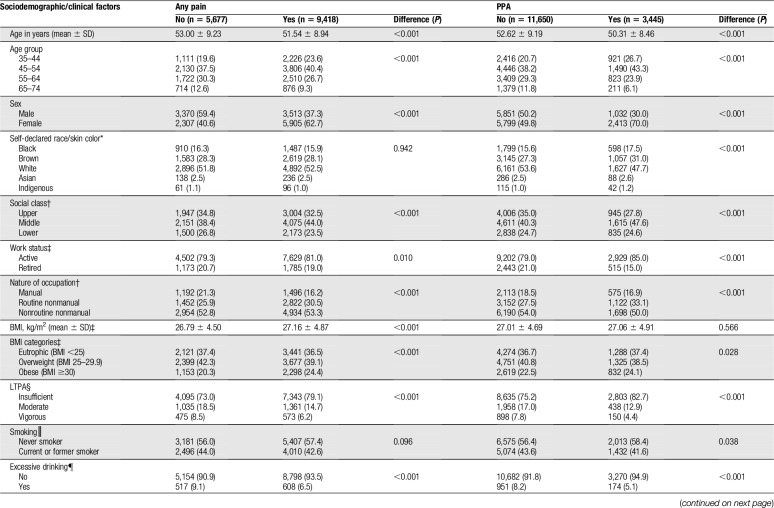

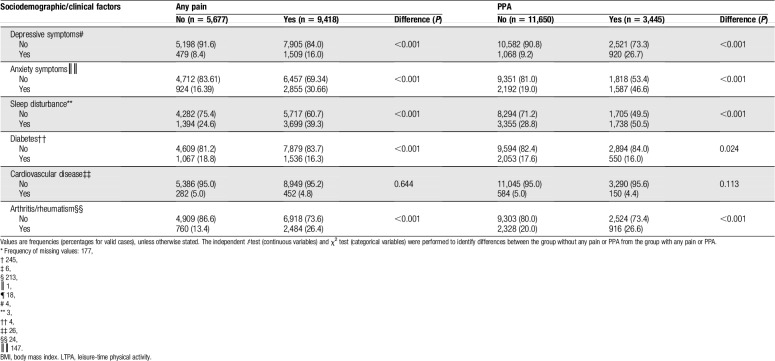
Associations between any pain or pain with psychological attributions (PPA) and sociodemographic/clinical factors (n = 15,095).

In the multivariable regression model, the following factors were associated with any pain: age (OR 0.97; 95% CI 0.97–0.98), female sex (OR 2.01; 95% CI 1.86–2.17), nonroutine nonmanual occupation (manual occupation as reference; OR 1.21; 95% CI 1.02–1.43), BMI (OR 1.01; 95% CI 1.00–1.02), moderate LTPA (insufficient LTPA as reference; OR 0.81; 95% CI 0.74–0.90), vigorous LTPA (insufficient LTPA as reference; OR 0.77; 95% CI 0.67–0.88), current/former smoking (OR 1.09; 95% CI 1.01–1.17), excessive drinking (OR 0.83; 95% CI 0.73–0.95), depressive symptoms (OR 1.28; 95% CI 1.13–1.44), anxiety symptoms (OR 1.63; 95% CI 1.48–1.79), sleep disturbance (OR 1.62; 95% CI 1.49–1.76), and arthritis/rheumatism (OR 2.18; 95% CI 1.98–2.41).

In the multivariable model with PPA as dependent variable, statistically significant associations were observed for age (OR 0.97; 95% CI 0.97–0.98), female sex (OR 1.86; 95% CI 1.69–2.05), moderate LTPA (insufficient LTPA as reference; OR 0.84; 95% CI 0.74–0.95), vigorous LTPA (insufficient LTPA as reference; OR 0.60; 95% CI 0.50–0.73), excessive drinking (OR 0.68; 95% CI 0.56–0.82), depressive symptoms (OR 1.96; 95% CI 1.75–2.20), anxiety symptoms (OR 2.45; 95% CI 2.23–2.69), sleep disturbance (OR 1.79; 95% CI 1.64–1.95), and arthritis/rheumatism (OR 1.32; 95% CI 1.19–1.46).

**Figure 2.**
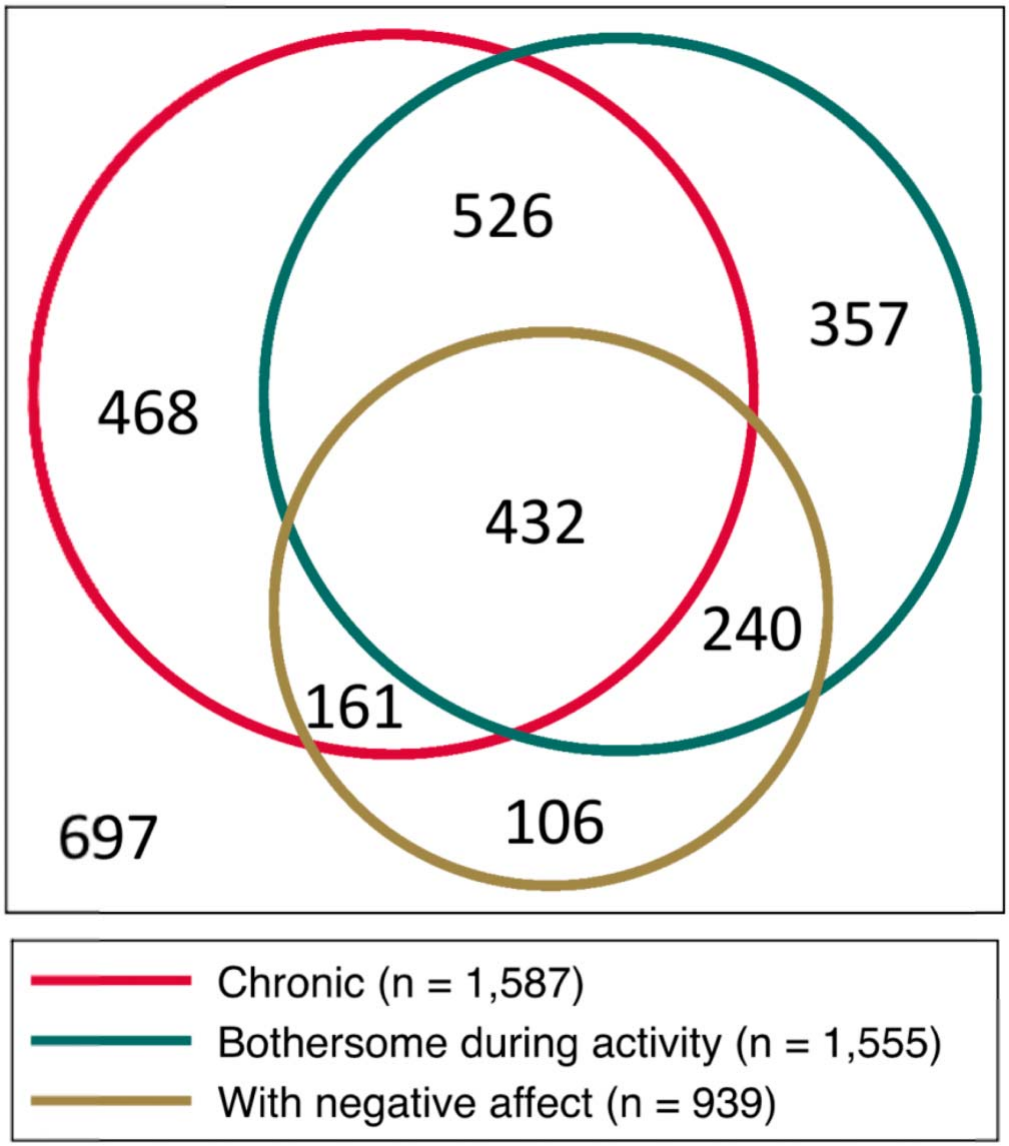
Area-proportional Venn diagram of the overlap among different characteristics of pain with psychological attributions—PPA (n = 2,988). Data on chronicity and bothersomeness were missing from one participant.

In the subsample with PPA, 58.6% had chronic pain, 57.4% perceived their pain as bothersome during activity and 34.7% had pain-related negative affect. Figure 2 shows the overlap observed among these multiple characteristics. The distribution of chronicity according to sociodemographic/clinical factors in the subsample with PPA and the results for univariable tests of association are presented in Table 3. Age, female sex, middle and lower social class (upper class as reference), nonmanual nature of occupation (manual occupation as reference), LTPA, depressive symptoms, anxiety symptoms, sleep disturbance, and arthritis/rheumatism were entered into the multivariable model, but none of them were independently associated with chronic pain. Regression outputs for multivariable analyses are described in the Supplementary material (available as supplemental digital content at http://links.lww.com/PR9/A58).

**Table 3:**
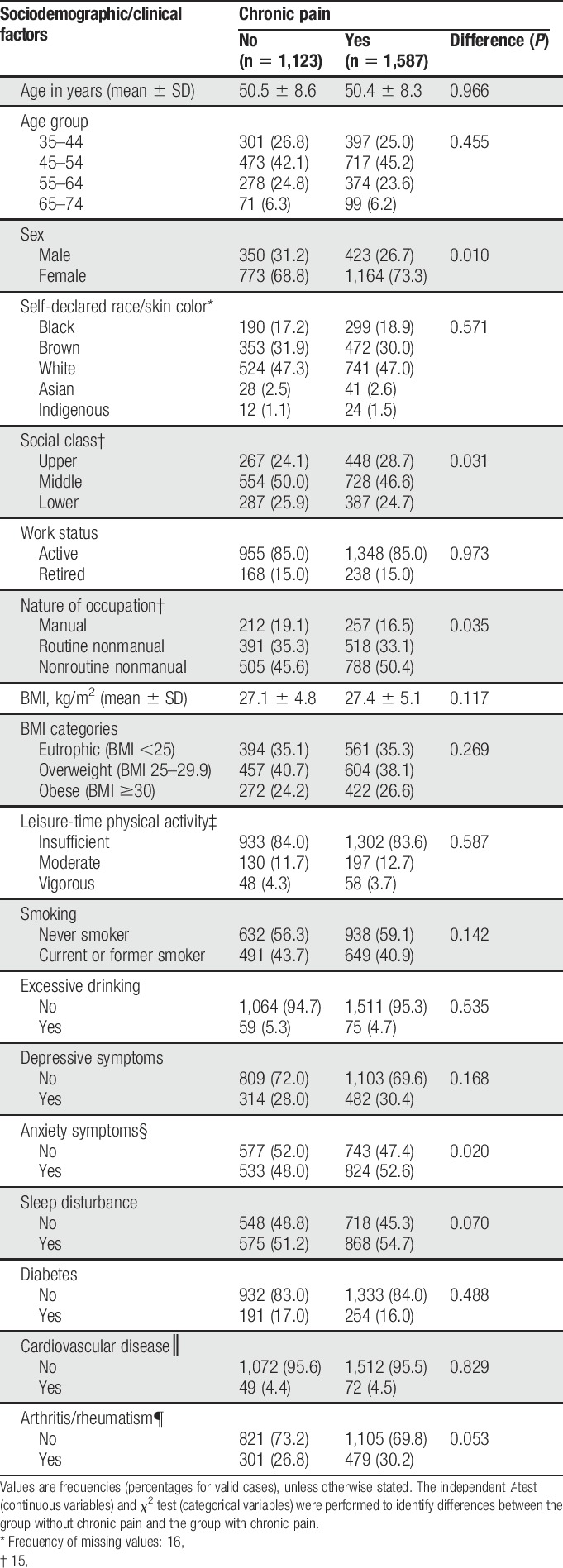
Association between the presence of chronic pain and sociodemographic/clinical factors in the subsample with pain with psychological attributions (PPA) (n = 2,710).

## 4. Discussion

The instrument used for the evaluation of common mental disorders in ELSA-Brasil included questions about the presence of pain, which allowed a preliminary investigation on the prevalence of pain and its associated factors at baseline of the cohort.

A large proportion (approximately two-thirds) of the individuals reported the presence of any pain in the past 30 days. The prevalence of pain in our study was somewhat superior to that found in a recent nationwide population-based telephone survey including 723 adults (mean age 41.2 years; 95% CI 39.7–42.7), where 42.0% of the surveyed individuals reported experiencing pain (any type) at the time of the interview or were currently taking pain medication.^54^ This might be explained by differences in the demographic composition between samples (eg, mean age in ELSA-Brasil was ∼10 years higher) and the use of point-prevalence pain assessments made over the telephone in the study of Souza et al.,^54^ compared with the face-to-face assessment of a 30-day period prevalence in ELSA-Brasil.

Nearly one-fourth of the included participants attributed their pain to psychological distress. The term “pain with psychological attributions” (PPA) has been previously used to denote somatic symptoms that, according to the person who experiences them, originate from a mental health problem.^53^ Semantically, this definition addresses only the self-perceived contribution of psychological factors to the experience of pain, thus distantiating itself from the current state-of-knowledge on the biopsychosocial nature of this condition.^25^ However, pain phenomena classified in our study under the PPA label may diverge from this simplistic interpretation and include conditions that potentially fulfill the criteria currently recommended by the International Association for the Study of Pain (IASP) for the diagnosis of chronic primary pain, as follows: pain that (1) persists/recurs for >3 months, (2) is associated with significant emotional distress and/or functional disability, (3) is not better accounted for by another diagnosis.^44^ Although our data cannot confirm whether those attributing their pain to psychological factors fulfilled criteria (3), this is somewhat supported by the convergence between IASP's chronic primary pain definition and the revised criteria for the classification of somatic symptom and related disorders (SSD, previously known as somatoform disorders), in the latest edition of the Diagnostic and Statistical Manual of Mental Disorders (DSM-5).^4,21^

The proportion of participants presenting PPA in our study (22.8%) was similar to that of adult Brazilians reporting chronic pain in previous population-based studies. For instance, in a telephone survey including 2,446 residents of São Paulo city, 28.1% of participants reported chronic pain (defined as pain ≥3 months).^34^ In addition, a nationally representative household survey including over 60 thousand Brazilians estimated the prevalence of chronic musculoskeletal conditions at 21.6% in 2013.^2^ However, a recent nationwide internet-based survey including an older sample of Brazilians (half of surveyed individuals were ≥65 years old) found a higher prevalence of chronic pain; eg, 64.2% reported pain with at least 6-month duration, and 76.2% considered their pain as “chronic, recurrent, or long-lasting.”^17^

To the best of our knowledge, 3 studies have previously investigated the prevalence of chronic pain among Brazilian civil servants from a university/research institute, and they also found higher prevalence estimates, ranging from 50% to 76%.^8,33,57^ The inclusion of smaller and less representative samples in these previous studies and the use of a less stringent definition of chronic pain (≥3 months) in the study of Barreto and Sá^8^ might have contributed to this difference. In another study including a representative sample of elderly municipal civil servants (aged >60 years) from all public sectors in a city in South Brazil, Dellaroza et al.^19^ found a 51% prevalence of pain ≥6 months. In other developing countries, investigations using civil servants as a model to study pain epidemiology are scarce, and we are unaware of studies investigating chronic pain in this target population. Studies conducted in Malaysia and Iran have reported an extremely high prevalence of pain of any duration among public service office workers, with 89%^36^ and 93%^37^ of workers experiencing musculoskeletal symptoms in the last 12 or 6 months, respectively.

We found univariable associations of any pain and PPA (any or chronic) with a vast list of sociodemographic and clinical characteristics. Multivariable analyses performed with data from the whole cohort suggest an independent role for factors previously shown to increase the likelihood of various pain conditions in other populations/settings (ie, female sex, insufficient physical activity, psychological distress, and arthritis). These analyses also showed an inverse association of age and excessive drinking with pain. Dionne et al.^22^ have previously demonstrated the presence of a linear relationship between severe pain and age but also a nonlinear and nonmonotonic relationship (U-shaped) between nonsevere (mild) pain and age; (ie, the prevalence of mild pain increases from young adulthood until the mid-fifties, but start to decrease after that age). Given the relaxed definition of pain used at baseline of ELSA-Brasil, it is possible that our pain estimates reflected mostly nonsevere problems, thus explaining the inverse association found in this study. The potentially protective effect of alcohol consumption on pain is also in line with findings from a high-quality longitudinal study among Danish twins^29^ as well as cross-sectional data considering previous month pain in older adults from 6 low- and middle-income countries.^1^

In the multivariable model investigating any type of pain, ELSA-Brasil participants with a nonmanual occupation had a higher chance of reporting pain than those with a manual occupation. Although this contrasts the widespread view on the deleterious effect of manual handling tasks for the musculoskeletal health, a recent systematic review could not find scientific support for a causal link between back pain and workplace manual handling.^49^ The lack of association between cardiovascular disease and chronic pain in the subsample with PPA also contrasts findings of a recent meta-analysis of population-based studies, which support the link between these conditions.^47^ This inconsistency is probably due to the exclusion of angina from the group of diagnoses considered under cardiovascular disease in our study, an approach than can provide less biased estimates by reducing the misclassification of individuals with angina-like chest pain of musculoskeletal origin (known as chest wall syndrome),^14^ or attributed to an anxiety disorder.^15^

Most sociodemographic/clinical factors associated with pain at baseline of ELSA-Brasil (2008–2010) were also found to be associated with doctor-diagnosed arthritis/rheumatism or self-reported spinal disorders in the 2013 Brazilian National Health Survey (PNAD, n = 60,202).^2^ However, an inconsistency was observed for moderate LTPA, which was found to reduce the odds of pain in ELSA-Brasil but to increase the likelihood of chronic musculoskeletal diseases in PNAD (when compared with insufficient physical activity/inactivity). A recently published meta-analysis of 36 prospective cohort studies has provided evidence to support the protective effect of moderate LTPA on the most prevalent pain condition worldwide, ie, low back pain.^52^

ELSA-Brasil is currently the largest epidemiological study conducted in Latin America investigating the development and progression of multiple NCDs and one of the few longitudinal cohorts performed in a non–high-income country to implement strategies of quality assurance and control that follow the same standards found in the most prominent cohorts of the developed world.^16,20,51^ Additional strengths of ELSA-Brasil include the prevention of biases that are common in survey research, such as selection and information bias. For instance, the study was deliberately planned as a cohort of civil servants to minimize losses to follow-up (ie, less than 6% of participants were lost after the first 4 years) and as a multicentric cohort including individuals living in major urban Brazilian cities with large and heterogeneous populations of mostly low- and middle-income levels.^6^

However, this introductory analysis on the epidemiology of pain in Brazil has some weaknesses. First, the instrument used to retrieve data on pain at baseline of ELSA-Brasil has limited our ability to provide more detailed information that could contribute to a deeper understanding of the structure, process, and outcome dimensions of pain in Brazil. For instance, we were not able to provide estimates for relevant pain phenotypes in the overall sample (eg, chronic pain instead of any pain), and for other clinical descriptors that could give an indication of the pathophysiology of the observed symptomatic episode (eg, nociceptive, neuropathic, and nociplastic pain). Second, measurement bias cannot be ruled out in the assessment of subjectively assessed exposures, including the diagnosis of cardiovascular diseases and arthritis/rheumatism. Finally, the study's cross-sectional design and the lack of consideration of the nature of the relationships among different factors included in multivariable analyses (eg, true confounders, colliders, or mediators) does not allow for causal interpretations of the reported associations.

An in-depth understanding of the epidemiology of chronic pain in Brazil is key for the development of adequate public health care policies as well as efficacious preventive and therapeutic strategies, which could reduce its personal and societal burden. Brazil is one of the world's key emerging economies, and where one of the fastest ageing populations and highest health care spending are currently taking place.^40^ Given the increased health care use among older Brazilians exhibiting chronic pain,^56^ the projected increase in the prevalence of this condition is likely to cause an important impact to the country's health care costs in the coming years. Although pain data were not collected during the second wave of assessments in ELSA-Brasil (except for the investigation of a selected group of musculoskeletal complaints in an ancillary study conducted at one of the 6 investigation centers^55^), future longitudinal analyses including data collected at the cohort's third wave (2017–2019) will allow for the investigation of the mechanisms underlying the development of multiple pain conditions in the Brazilian population, including chronic, disabling, and widespread/generalized pain.

## Disclosures

The authors have no conflict of interest to declare.

S.M. Barreto is a research fellow of CNPq and Foundation for Research Support of the State of Minas Gerais (FAPEMIG). The investigation was performed while L.A.C. Machado was a Postdoctoral Fellow supported by CAPES-BR.

## Appendix A. Supplemental digital content

Supplemental digital content associated with this article can be found online at http://links.lww.com/PR9/A58.

## Supplementary Material

SUPPLEMENTARY MATERIAL
