# Supplementary material for: Prevalence of pain and associated factors in Brazilian civil servants: an introductory analysis using baseline data from the ELSA-Brasil cohort
Source: Pain Rep. 2019 Dec 6;4(6):e797. doi: 10.1097/PR9.0000000000000797 (PMC6903374; doi:10.1097/PR9.0000000000000797)
Supplement: SUPPLEMENTARY MATERIAL [file pr9-4-e797-s001.docx]

**SUPPLEMENTARY MATERIAL**

Prevalence of pain and associated factors in Brazilian civil servants: An introductory analysis using baseline data from the ELSA-Brasil cohort

*Pain Reports*

Luciana A C Machado, Rosa W Telles, Isabela Benseñor, Sandhi M Barreto

Corresponding author: Dr. Luciana A Machado. Faculty of Medicine, Universidade Federal de Minas Gerais, Avenida Alfredo Balena n. 110, Funcionários, Belo Horizonte, MG 30130-100, Brazil. Email: [machadolac@hotmail.com](mailto:machadolac@hotmail.com)

**Supplementary Table 1.** Multivariable logistic regression model investigating the association between sociodemographic/clinical factors and any pain (n=14,443).

|  | **Odds Ratio** | **Std. Err.** | **95% Confidence Interval** | **P** |
| --- | --- | --- | --- | --- |
| Age | 0.97 | 0.00 | 0.97-0.98 | **< 0.001** |
| Female sex | 2.01 | 0.08 | 1.86-2.17 | **< 0.001** |
| Social class (ref. upper) |  |  |  |  |
| Middle | 0.99 | 0.05 | 0.89-1.10 | 0.809 |
| Lower | 0.93 | 0.08 | 0.79-1.10 | 0.389 |
| Active work status | 1.05 | 0.06 | 0.93-1.18 | 0.422 |
| Nature of occupation (ref. manual) |  |  |  |  |
| Routine non-manual | 1.10 | 0.08 | 0.95-1.28 | 0.197 |
| Non-routine non-manual | 1.21 | 0.10 | 1.02-1.43 | **0.028** |
| BMI | 1.01 | 0.00 | 1.00-1.02 | **0.002** |
| LTPA (ref. insufficient) |  |  |  |  |
| Moderate | 0.81 | 0.04 | 0.74-0.90 | **< 0.001** |
| Vigorous | 0.77 | 0.05 | 0.67-0.88 | **< 0.001** |
| Smoking | 1.09 | 0.04 | 1.01-1.17 | **0.031** |
| Excessive drinking | 0.83 | 0.06 | 0.73-0.95 | **0.008** |
| Depressive symptoms | 1.28 | 0.08 | 1.13-1.44 | **< 0.001** |
| Anxiety symptoms | 1.63 | 0.08 | 1.48-1.79 | **< 0.001** |
| Sleep disturbance | 1.62 | 0.07 | 1.49-1.76 | **< 0.001** |
| Diabetes | 0.93 | 0.05 | 0.84-1.03 | 0.145 |
| Arthritis/rheumatism | 2.18 | 0.11 | 1.98-2.41 | **< 0.001** |

BMI, body mass index. LTPA, leisure-time physical activity.

**Supplementary Table 2.** Multivariable logistic regression model investigating the association between sociodemographic/clinical factors and pain with psychological attributions (PPA) (n=14,277).

|  | **Odds Ratio** | **Std. Err.** | **95% Confidence Interval** | **P** |
| --- | --- | --- | --- | --- |
| Age | 0.97 | 0.00 | 0.97-0.98 | **< 0.001** |
| Female sex | 1.86 | 0.09 | 1.69-2.05 | **< 0.001** |
| Self-declared race/skin color (ref. Black) |  |  |  |  |
| Brown | 1.12 | 0.07 | 0.99-1.27 | 0.080 |
| White | 0.96 | 0.06 | 0.85-1.08 | 0.493 |
| Asian | 1.04 | 0.15 | 0.78-1.39 | 0.770 |
| Indigenous | 1.28 | 0.27 | 0.85-1.93 | 0.232 |
| Social class (ref. upper) |  |  |  |  |
| Middle | 1.07 | 0.07 | 0.95-1.21 | 0.248 |
| Lower | 1.02 | 0.10 | 0.85-1.23 | 0.829 |
| Active work status | 0.87 | 0.06 | 0.75-1.01 | 0.061 |
| Nature of occupation (ref. manual) |  |  |  |  |
| Routine non-manual | 0.96 | 0.09 | 0.80-1.14 | 0.637 |
| Non-routine non-manual | 1.03 | 0.10 | 0.85-1.26 | 0.745 |
| BMI | 0.99 | 0.00 | 0.98-1.00 | 0.082 |
| LTPA (ref. insufficient) |  |  |  |  |
| Moderate | 0.84 | 0.05 | 0.74-0.95 | **0.005** |
| Vigorous | 0.60 | 0.06 | 0.50-0.73 | **< 0.001** |
| Smoking | 1.00 | 0.04 | 0.91-1.09 | 0.913 |
| Excessive drinking | 0.68 | 0.06 | 0.56-0.82 | **< 0.001** |
| Depressive symptoms | 1.96 | 0.11 | 1.75-2.20 | **< 0.001** |
| Anxiety symptoms | 2.45 | 0.12 | 2.23-2.69 | **< 0.001** |
| Sleep disturbance | 1.79 | 0.08 | 1.64-1.95 | **< 0.001** |
| Diabetes | 1.03 | 0.06 | 0.91-1.16 | 0.625 |
| Arthritis/rheumatism | 1.32 | 0.07 | 1.19-1.46 | **< 0.001** |

BMI, body mass index. LTPA, leisure-time physical activity.

**Supplementary Table 3.** Multivariable logistic regression model investigating the association between sociodemographic/clinical factors and chronic pain in the subsample with pain with psychological attributions (PPA) (n=2,595).

|  | **Odds Ratio** | **Std. Err.** | **95% Confidence Interval** | **P** |
| --- | --- | --- | --- | --- |
| Age | 1.0 | 0.00 | 0.99-1.01 | 0.596 |
| Female sex | 1.15 | 0.11 | 0.96-1.38 | 0.122 |
| Social class (ref. upper) |  |  |  |  |
| Middle | 0.83 | 0.10 | 0.66-1.05 | 0.119 |
| Lower | 1.00 | 0.18 | 0.71-1.43 | 0.980 |
| Nature of occupation (ref. manual) |  |  |  |  |
| Routine non-manual | 1.17 | 0.19 | 0.85-1.61 | 0.322 |
| Non-routine non-manual | 1.36 | 0.25 | 0.95-1.96 | 0.091 |
| LTPA (ref. insufficient) |  |  |  |  |
| Moderate | 1.09 | 0.14 | 0.86-1.40 | 0.480 |
| Vigorous | 0.91 | 0.19 | 0.60-1.36 | 0.641 |
| Depressive symptoms | 1.02 | 0.10 | 0.85-1.23 | 0.818 |
| Anxiety symptoms | 1.12 | 0.10 | 0.95-1.32 | 0.188 |
| Sleep disturbance | 1.09 | 0.09 | 0.93-1.29 | 0.278 |
| Arthritis/rheumatism | 1.19 | 0.11 | 0.99-1.42 | 0.064 |

LTPA, leisure-time physical activity.
